# Behavior change in a lifestyle intervention for type 2 diabetes prevention in Dutch primary care: opportunities for intervention content

**DOI:** 10.1186/1471-2296-14-78

**Published:** 2013-06-07

**Authors:** Paulina WA Vermunt, Ivon EJ Milder, Frits Wielaard, Caroline A Baan, Jos DM Schelfhout, Gert P Westert, Hans AM van Oers

**Affiliations:** 1 Scientific Centre for Transformation in Care and Welfare (Tranzo), University of Tilburg, Tilburg, the Netherlands; 2 Centre for prevention and health services research, National Institute for Public Health and the Environment, Bilthoven, the Netherlands; 3 Association of primary care practices ‘De Ondernemende Huisarts’ (DOH), Eindhoven, the Netherlands; 4 Scientific Institute for Quality of Healthcare (IQ Healthcare), Radboud University Nijmegen Medical Centre, Nijmegen, the Netherlands; 5 Department of public health status and forecasts, National Institute for Public Health and the Environment, Bilthoven, the Netherlands

**Keywords:** Type 2 diabetes, Primary care, Behavior change, Lifestyle intervention

## Abstract

**Background:**

Despite the favorable effects of behavior change interventions on diabetes risk, lifestyle modification is a complicated process. In this study we therefore investigated opportunities for refining a lifestyle intervention for type 2 diabetes prevention, based on participant perceptions of behavior change progress.

**Methods:**

A 30 month intervention was performed in Dutch primary care among high-risk individuals (FINDRISC-score ≥ 13) and was compared to usual care. Participant perceptions of behavior change progress for losing weight, dietary modification, and increasing physical activity were assessed after18 months with questionnaires. Based on the response, participants were categorized as ‘planners’, ‘initiators’ or ‘achievers’ and frequencies were evaluated in both study groups. Furthermore, participants reported on barriers for lifestyle change.

**Results:**

In both groups, around 80% of all participants (intervention: N = 370; usual care: N = 322) planned change. Except for reducing fat intake (p = 0.08), the number of initiators was significantly higher in the intervention group than in usual care. The percentage of achievers was high for the dietary and exercise objectives (intervention: 81–95%; usual care: 83–93%), but was lower for losing weight (intervention: 67%; usual care: 62%). Important motivational barriers were ‘*I already meet the standards*’ and ‘*I*’*m satisfied with my current behavior*’. *Temptation to snack*, *product taste* and *lack of time* were important volitional barriers.

**Conclusions:**

The results suggest that the intervention supports participants to bridge the gap between motivation and action. Several opportunities for intervention refinement are however revealed, including more stringent criteria for participant inclusion, tools for (self)-monitoring of health, emphasis on the ‘small-step-approach’, and more attention for stimulus control.

**Trial registration:**

Netherlands Trial Register: NTR1082

## Background

Type 2 diabetes mellitus is a serious illness, leading to severe complications [1] and increased mortality [2]. Global incidence of the disease is estimated to rise to 552 million individuals in 2030, posing a great burden to many countries worldwide [3]. Behavior change interventions can however prevent or delay development of type 2 diabetes in individuals at high risk [4]. In the Diabetes Prevention Study (DPS) and the Diabetes Prevention Program (DPP) for example, dietary improvement and more physical activity led to a reduction in diabetes incidence of nearly 60% in 4 years [5,6].

Despite the favorable effects of behavior change interventions on type 2 diabetes risk, lifestyle modification is a complicated process [7,8]. Furthermore, due to organizational and financial barriers, translation of successful lifestyle interventions into daily life settings is challenging [9,10]. More insight into the process of behavior change may reveal opportunities for refining intervention content and may thereby potentially improve intervention effectiveness [9-11]. Nevertheless, evaluation of behavior change in diabetes prevention programs remains limited [10].

The ‘Active Prevention in High Risk individuals Of Diabetes Type 2 in and around Eindhoven’ (APHRODITE) study investigates the effectiveness and feasibility of lifestyle counseling for diabetes prevention in Dutch primary care. In this article we investigate the perceived behavior change phase for several lifestyle objectives of participants receiving lifestyle counseling and receiving usual care. In addition, we assess the main perceived barriers for planning or achieving behavior change. Based on these insights we discuss opportunities for refining intervention content.

## Methods

Participants were recruited in January 2008 by 48 general practitioners (GPs) and 24 nurse practitioners from 14 primary care practices in the Netherlands. A Dutch translation of the Finnish FINDRISC [12] was sent to GP patients aged ≥40 and ≤70 years. All individuals with a score ≥13 points (n = 1533) were invited to participate [13]. To minimize selection bias, individuals were at random allocated to either the intervention group (n = 479) or the usual care group (n = 446). Randomization was performed on the level of the individual [13]. Details of participant recruitment, randomization and intervention reach were described previously [13]. Individuals who were diagnosed with diabetes during the follow-up were excluded from the study and were referred to the GP for further care.

### Intervention group

**Figure 1:**
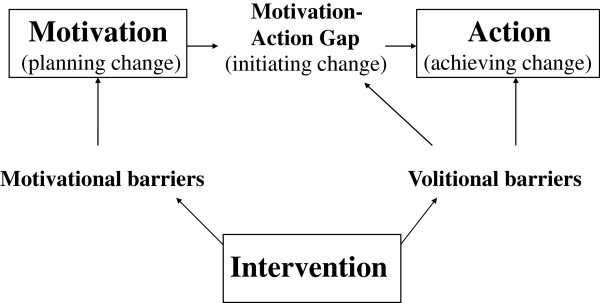
**Intervention effects on participant behavior change.** During the process of behavior change, individuals progress from a motivational phase (planning change), via the motivation-action gap (initiating change) towards an action phase (achieving change). Progress through the different phases is limited by motivational and volitional barriers, which can be affected using lifestyle counseling.

The APHRODITE intervention was based on the transtheoretical model [7] and was designed to support participant progress from a motivational phase (planning change), via the motivation-action gap (initiating change) towards an action phase (achieving change) (Figure 1). Progress through the phases is limited by motivational and volitional barriers, which can be influenced by lifestyle counseling. In our study, a combination of behavior change techniques was used (motivational interviewing, filling out decisional balance sheets, goal setting, developing action plans, barrier identification, relapse prevention) [8,14]. Details of the theoretical framework of the APHRODITE intervention are described in Additional file 1.

After the admission interview with the GP [13], 11 consultations of 20 minutes were scheduled over 30 months with alternately the nurse practitioner and the GP (Additional file 2). In addition, 5 group meetings were organised by dieticians and physiotherapists to provide more detailed information on diet and exercise. Moreover, intervention-group participants were invited for a 1-hour consultation with a dietician, in which a 3-day food record was discussed.

Five project objectives were specified: weight reduction of at least 5% if overweight, physical exercise of moderate to high intensity for at least 30 minutes a day for at least five days a week, dietary fat intake less than 30% and saturated fat intake less than 10% of total energy intake and dietary fibre intake of at least 3.4 g per MJ. Following a tailor-made and small-step approach [15], participants were however stimulated by the nurse practitioner to set individual (intermediate) goals and develop individual action plans.

The programme was free of charge for all participants. Providers received financial reimbursement for all consultations with their participants according to Dutch payment standards. The intervention was registered with the Dutch Trial Register (NTR1082). The Medical Ethical Review Committee of the Catharina Hospital in Eindhoven gave ethical approval to the study (M07-1705). All participants gave informed consent for participation.

### GP and nurse practitioner training

Before the start of the study, all GPs and nurse practitioners received a two-evening directive instruction on the theoretical framework of the intervention and its translation into practice (the content of this instruction is summarized in Additional file 1 and the mode of delivery in Additional file 3). In addition, a manual with all topics discussed and key-message cards for use during consultations were sent both on paper and by email. Moreover, as they intensively guided the behavior change process, all nurse practitioners received a five-evening course in motivational interviewing (MI) [16] (briefly summarized in Additional file 3). As a part of the course, active role-playing was performed and consultations with participants were audio-taped for feedback purposes. During the study, regular return-meetings were organised with GPs (once a year) and nurse practitioners (every half a year).

### Usual care group

During the admission interview, participants in the usual care group received oral and written information about type 2 diabetes and a healthy lifestyle. The nurse practitioner was visited only for measurements at baseline and after 6, 18 and 30 months. Apart from the admission interview participants did not have study-related encounters with the GP.

### Participant questionnaires

Questionnaires were filled out after 18 months of intervention (Additional file 4). Response was 92% in the intervention group and 85% in the usual care group. To reduce detection bias, individuals were not made aware of being in the intervention or the usual care group; they were only told to be in either of two groups with different contact frequency.

To gain more insight into perceived behavior change progress (Figure 1), participants were first asked whether they were planning to change a behavior or were already acting on it. When answering *yes*, they were asked whether they had initiated change. When answering *yes*, they were asked whether they had achieved change. For analysis, all participants who indicated to have planned, but not initiated change were called *planners*. All who reported to have initiated, but not achieved change were called *initiators*. Participants who indicated to have achieved change were called *achievers*.

Motivational and volitional barriers for behavior change were inquired for all lifestyle objectives using open questions. Motivational barriers were collected from non-planners with reporting rates ranging from 87% to 99% (intervention) and from 83% to 99% (usual care). Volitional barriers were collected from initiators and achievers, with reporting rates ranging from 81% to 88% (intervention) and from 80% to 94% (usual care). All barriers were coded by the main investigator and two research assistants; inconsistencies were checked by the main investigator. Categorization of the barriers was based on frameworks developed by Penn et al. [11] and Grol and Wensing [17].

### Sample size calculation

Sample size calculation was based on the main outcome diabetes incidence. As implementation of lifestyle interventions in real life settings is challenging [9,10], modest differences between groups were expected. To detect small differences in diabetes incidence (Cohen’s conventional effect size of 0.1), with a power of 0.8, 393 individuals were needed in each arm. As in total 925 individuals could be included, this allowed for a dropout rate of approximately 15%, which was in line with others [4]. Post-hoc power analysis showed that the power to detect small differences between groups in the percentage of planners was 0,75 for all lifestyle objectives. For a difference in initiators, the power ranged between 0,63-0,68 and for a difference in achievers between 0,46-0,59.

### Statistical analysis

Differences between study groups were analysed with chi-square tests using SPSS 18.0. A p-value of <0.05 was considered significant. Additionally, the effect of a bonferroni adjustment for multiple comparisons was investigated (p = 0.05/15 = <0.003). Individuals who developed diabetes during follow-up intervention: N = 32 (6.8%); usual care: N = 32 (7.3%) or who ended participation intervention: N = 46, 9.6%; usual care: N = 36 (8.1%) were excluded from the study and from analysis.

## Results

No significant differences in baseline characteristics (sex, age, education, FINDRISC-score, smoking) and clincial measures (mean bmi, mean fasting and 2-hour glucose values) were observed between study groups. Table 1 shows perceived behavior change at 18 months of participants in both groups for all lifestyle objectives. The percentage of planners ranged from 76% to 85% in both groups and was comparable between groups. The percentage of initiators ranged from 72% to 86% (intervention) and from 59% to 79% (usual care). Except for reducing fat intake (p = 0.08), the percentage of initiators was significantly higher in the intervention group than in the usual care group. When a bonferroni adjustment was applied significance was lost for all objectives. For the nutrition and physical activity objectives, the percentage of achievers ranged from 81% to 95% (intervention) and from 83% to 93% (usual care). For losing weight these percentages were 67% (intervention) and 62% (usual care). For all lifestyle objectives, the percentage of achievers did not significantly differ between groups.

**Table 1:** Perceptions of participants in both study groups of behavior change phase (planning, initiating or achieving change) at 18 months for five lifestyle objectives

**Objective**	**Group**	Planned change*	Initiated change*	Achieved change*
		**(% of total (N))**	**(% of planners (N))**	**(% of initiators (N))**
Lose weight	I	81 (300)	83 (248)	67 (167)
	UC	82 (264)	75 (197) **	62 (122)
Increase dietary fibre intake	I	76 (279)	72 (198)	87 (172)
	UC	77 (245)	59 (144) **	90 (130)
Reduce total fat intake	I	83 (309)	85 (260)	95 (246)
	UC	83 (273)	79 (216)	93 (200)
Reduce saturated fat intake	I	85 (319)	84 (259)	93 (240)
	UC	85 (277)	77 (211) **	92 (194)
Increase physical activity	I	81 (303)	84 (250)	81 (202)
	UC	76 (248)	74 (180) **	83 (149)

Abbreviations: *I* intervention, *UC* usual care.

* Drop-outs and individuals diagnosed with type 2 diabetes during follow-up were left out of analysis.

** Significant differences between groups as tested by chi-square tests (p < 0.05). Significance was lost after Bonferonni adjustment for multiple comparisons (p < 0.05: 15 = <0.003).

Table 2 summarizes the most-mentioned behavior change barriers of participants in both groups. Both the motivational and volitional barriers were highly comparable between the study groups. For all objectives an important barrier for planning change was ‘*I already meet the standards*’. This especially applied to the dietary fibre, total fat and physical activity objectives, with reporting-rates ranging from 56 to 66% (intervention) and from 48 to 69% (usual care). Another important factor limiting participant motivation was ‘*I*’*m satisfied with my health and*/*or behavior*’, especially regarding weight (intervention: 26%; usual care: 35%).

**Table 2:** Top-three barriers for planning or achieving behavior change of participants in both study groups for five lifestyle objectives

**Motivational barriers ‡**	**Intervention group**	**N (%)** *	**Usual care group**	**N (%)** *
Weight loss	1. Weight is healthy	29 (40)	1. Weight is healthy	24 (35)
	2. Satisfied with weight	19 (26)	2. Satisfied with weight	24 (35)
	3. Achieved my goals	5 (7)	3. Achieved my goals	3 (4)
Increase dietary fibre intake	1. Eat enough dietary fibre	59 (60)	1. Eat enough dietary fibre	49 (54)
	2. Satisfied with what I eat	10 (10)	2. Satisfied with what I eat	9 (10)
	3. Already took dietary fibre into account in diet	5 (5)	3. Already took dietary fibre into account in diet	8 (9)
Reduce fat intake	1. Diet does not contain too much fat	44 (56)	1. Diet does not contain too much fat	29 (48)
	2. Already took fat intake into account in diet	12 (15)	2. Already took fat intake into account in diet	14 (23)
	3. Satisfied with what I eat	4 (5)	3. Satisfied with health	6 (10)
Reduce saturated fat intake	1. Diet does not contain too much saturated fat	23 (37)	1. Already took saturated fat into account in diet	16 (28)
	2. Already took saturated fat into account in diet	14 (23)	2. Diet does not contain too much saturated fat	9 (16)
	3. Satisfied with what I eat	6 (10)	3. Lack of knowledge	8 (14)
Increase physical exercise	1. Have enough exercise	55 (66)	1.Have enough exercise	68 (69)
	2. Physical inabilities	16 (19)	2. Physical inabilities	18 (18)
	3. Not enough time	3 (4)	3. Not enough time	3 (3)
**Volitional barriers ‡**	**Intervention group**	**N (%)**	**Usual care group**	**N (%)**
Weight loss	1. Temptation to snack	51 (26)	1. Temptation to snack	36 (21)
	2. Continuity, relapse **	26 (13)	2. Continuity, relapse **	23 (14)
	3. Special occassions	21 (11)	3. Special occassions	21 (12)
Increase dietary fibre intake	1. No difficulties	84 (52)	1. No difficulties	77 (67)
	2. Taste of products	23 (14)	2. Taste of products	9 (8)
	3. Product knowledge	11 (7)	3. Product knowledge	6 (5)
Reduce fat intake	1. Temptation to snack	69 (32)	1. No difficulties	62 (33)
	2. No difficulties	64 (29)	2. Temptation to snack	54 (28)
	3. Taste of products	34 (16)	3. Taste of products	28 (15)
Reduce saturated fat intake	1. No difficulties	75 (33)	1. No difficulties	64 (38)
	2. Temptation to snack	44 (19)	2. Temptation to snack	31 (18)
	3. Taste of products	31 (14)	3. Taste of products	23 (14)
Increase physical exercise	1. No difficulties	45 (22)	1. Not enough time	39 (23)
	2. Not enough time	35 (17)	2. No difficulties	30 (18)
	3. Continuity, relapse **	26 (12)	3. Continuity, relapse **	23 (14)

‡ Motivational barriers were collected from non-planners; volitional barriers from initiators and achievers.

* Drop-outs and individuals diagnosed with type 2 diabetes during follow-up were left out of analysis.

** ‘Continuity’ is defined as ‘maintaining a new healthy habit on the longer term’.

For the weight loss and physical activity objectives, continuity (maintaining a new habit on the longer term) was an often-reported bottleneck (intervention: 13% and 12%; usual care: 14% for both). For the weight loss and fat-related objectives, temptation to snack was an important volitional barrier, with reporting-rates ranging from 19% to 32% (intervention) and from 18% to 28% (usual care). Lack of time was a bottleneck for increasing physical activity (intervention: 17%; usual care: 23%). A substantial number reported ‘no difficulties’ when trying to achieve dietary objectives (intervention: 33%-52%; usual care: 33% to 67%).

## Discussion

Although lifestyle change can lead to reduced diabetes risk, it is a complicated process [7,8]. In this article we therefore investigated the perceived behavior change phase for several lifestyle objectives of participants receiving lifestyle counseling and receiving usual care. In addition, we assess the main perceived barriers for planning or achieving behavior change. Based on these insights we discuss opportunities for refining intervention content (Table 3).

**Table 3:** Opportunities for refining intervention content based on participant perceptions of behavior change progress

**Phase**	**Finding**	**Explanation / interpretation**	**Opportunity for intervention refinement**
Motivation(planning change)	‘*I already meet the standards’* and ‘*I’m satisfied with my health/ behavior’* are important motivational barriers	Inclusion of participants with a relatively healthy lifestyle, limiting motivation to change [10,19]	Increase FINDRISC-value for participant inclusion or additional evaluation of lifestyle prior to invitation
		Inability of participants to correctly interpret their lifestyle	Better inform participants about the standards reflecting healthy lifestyle
			Introduction of tools for (self)-monitoring of health and lifestyle [21]
Motivation-Action Gap (initiating change)	Significant differences in the number of initiators between study groups for nearly all objectives	The intervention seems to help participants bridge the gap between motivation and action [8,22]	Continue to stimulate participants to set goals and to develop concrete action plans [8,14,22]
	A substantial part of the planners do not put their plans into action	Lack of action self-efficacy of non-initiators [23]	Underline the small-step approach of the intervention [15]
Action (achieving change)	A majority of initiators reports to have achieved change for diet and physical activity, AND Large numbers of initiators reported no difficulties achieving change, BUT Modest risk factor reductions [25]	Too optimistic perceptions of participants of lifestyle change success.	Introduction of tools for (self)-monitoring for parti-cipants to reflect on behavior change progress [21]
			Guard participant progress towards achieving the project objectives
			Provide GPs and nurse practitioners with tools for monitoring participant progress
	*Continuity* (maintaining a new habit on the longer term) is an important barrier for losing weight and increasing physical activity.	Tendency of participants to make too drastic alterations in the lifestyle, easily resulting in relapse [15].	Following the small-step approach: stimulate participants to set intermediate goals [15]
			Keep a goal and performance logbook to facilitate continuous evaluation of participant progress [21]
	*Resisting temptation to snack* is an often-mentioned difficulty for the weight loss and dietary objectives.	Participants may have difficulties to control internal and external stimuli [27]	Encourage to avoid cues [27]
			Stimulate to engage social support [14,26]
			Support participants to monitor circumstances of habitual behavior to identify future high-risk situations and beforehand develop strategies [21]

### The motivational phase (planning change)

Participation in a behavior change program implies that individuals are motivated to improve their lifestyle [18]. In line with this hypothesis, the percentage of non-planners was low in both groups for all objectives. Two important barriers for planning change were the conviction that recommendations were already met and satisfaction with the current behavior. The relatively low cut-off-value of the FINDRISC (≥13 points) may have led to the selection of individuals with a relatively healthy lifestyle, limiting the motivation to change [10,19]. In line with this hypothesis, 40% of the non-planners had a healthy BMI (<25 kg/m^2^) at 18 months versus 13% of the planners (p = <0.0001). An increase in the FINDRISC-value for inclusion or evaluation of participant lifestyle prior to invitation are therefore recommended.

Another explanation for the large number of non-planners convinced of their health may be an inability of participants to correctly interpret the lifestyle. For the dietary objectives for example, 58% to 87% of the convinced non-planners incorrectly thought they already met the recommendations. Non-planners should therefore be better informed about the standards reflecting a healthy lifestyle. Second, introduction of tools for self-monitoring, like in the PRAEDIAS-study [20,21] may help participants reflect on their health.

#### The motivation-action gap (initiating change)

Significant differences in the number of initiators were observed between the groups for nearly all objectives. This result suggests that the intervention was successful in helping participants bridge the gap between motivation and action. Overcoming this gap is regarded as an important step in behavior change [8,22]. Possibly contributing to taking this step, participants were stimulated to set goals and develop concrete action plans [8,14,22]. Despite this apparant success, 15% to 28% of the planners in the intervention group did not put their plans into action. This may partially be explained by a lack of action self-efficacy [23]. Action self-efficacy could potentially be enlarged by underlining the ‘small-step-approach’ of the intervention, in which participants are encouraged to make small, but meaningful changes that can more easily be sustained long-term [15].

The differences in the number of initiators were no longer significant after applying a bonferroni adjustment for multiple comparisons. This loss of significance could however partially be explained by a lack of statistical power to detect small differences in the number of initiators between groups (0,63-0,68). Because behavior change phase was not assessed at baseline we cannot exclude that the difference in the percentage of initiators between groups may partially be due to baseline differences. However, as persons were randomly assigned to either group and no differences in baseline characteristics or clinical measures were observed, this possibility seems unlikely. The absence of baseline differences between groups also makes it unlikely that the results were subject to selection bias. Intervention group participants may however have been aware of the high(er) contact frequency with the GP and the nurse practitioner and may therefore more easily have given socially desirable answers (detection bias).

#### The action phase (achieving change)

A majority of initiators in the intervention group indicated to have achieved change regarding diet and physical activity. Risk factor reductions shown in the intervention group after 18 months however were modest (BMI: -0.1 kg/m^2^, p = 0.66; fasting glucose: -0.02 mmol/l, p = 0.77) and no significant difference in diabetes incidence was found between the intervention group (10.0%) and the usual care group (11.9%) (p = 0.99) after 30 months [24,25]. This discrepancy may be explained by the inability of participants to correctly monitor lifestyle change. Introduction of tools for self-monitoring may therefore also be recommended for participants to reflect on their progress. In other studies, self-monitoring was also found to contribute to lifestyle change [14,20,26]. Too optimistic perceptions of initiators may also have been caused by setting goals that were not challenging enough. It is therefore important that providers guard progress towards achieving the project objectives.

Despite the positive view of initiators, several barriers for achieving lifestyle change were reported. For the weight loss and physical activity objectives, continuity (maintaining a new habit on the longer term) was an often-reported bottleneck. This result may reflect the tendency to make too drastic alterations in the lifestyle (extreme dieting, intensive work-outs), that can easily result in relapse [15]. Following the small-step approach of our intervention [15], participants should therefore be stimulated to set intermediate goals. In addition, a goal and performance logbook may facilitate continued evaluation of participant progress [21].

For the weight loss and dietary objectives, resisting temptation to snack was an often-mentioned difficulty. This result underlines the importance of techniques to control internal and external stimuli, as described by for example Shaw et al. [27]. Professionals may support stimulus control by encouraging participants to avoid cues (for example not have snacks stored at home) [27] and to engage social support [14,26]. In addition, monitoring of psychological causes for and circumstances of habitual behavior [21] may help participants to identify future high-risk situations, so that strategies can be developed beforehand.

#### Strengths and limitations

Our study provides more insight into the black box between the intervention on the one side and its effectiveness of the other side. The high response rates make it unlikely that missing values have markedly influenced the results. When answering questions, participants may however have been affected by recent experiences. The missing data of drop-outs and individuals diagnosed with diabetes may have influenced participant outcomes. In addition, as barriers to change were only inquired once, development over time could not be investigated. An opposite approach of inquiring facilitators for change could provide valuable additional insights into behavior change. Furthermore, it would have been useful to provide participants with the opportunity to express their views and preferences during intervention development.

## Conclusions

A better insight into the process of behavior change can contribute to better adapted and potentially more effective interventions for diabetes prevention [9-11]. Although the results suggest that the APHRODITE intervention helps participants bridge the gap between motivation and action, several opportunities for refining intervention content are revealed. Recommendations for practice include an increase in the FINDRISC value for participant inclusion, instruction about standards reflecting a healthy lifestyle, tools for (self)-monitoring of health and lifestyle, goal setting and action planning, engaging social support, monitoring of causes for and circumstances of habitual behavior, a larger emphasis on the small-step-approach, and more attention for controlling environmental and psychological stimuli.

## Competing interests

All authors declare that they have no competing interests.

## Authors’ contributions

PV contributed to the study design, to the acquisition of the data, and to analysis and interpretation of the data and drafted the manuscript. IM, FW, CB, JS, GW and HvO contributed to the study design and to the interpretation of the data and critically revised the manuscript. All authors read and approved the final manuscript.

## Pre-publication history

The pre-publication history for this paper can be accessed here:

http://www.biomedcentral.com/1471-2296/14/78/prepub

## Supplementary Material

Additional file 1 Overview of the theoretical framework of the APHRODITE intervention to support behavior change. Click here for file

Additional file 2 Planning of the APHRODITE intervention and content of the group consultations. Click here for file

Additional file 3 Mode of delivery of the GP and nurse practitioner training. Click here for file

Additional file 4 Questionnaire behavioral change APHRODITE study. Click here for file
