# Supplementary material for: Behavior change in a lifestyle intervention for type 2 diabetes prevention in Dutch primary care: opportunities for intervention content
Source: BMC Fam Pract. 2013 Jun 7;14:78. doi: 10.1186/1471-2296-14-78 (PMC3706294; doi:10.1186/1471-2296-14-78)
Supplement: Additional file 1 — Overview of the theoretical framework of the APHRODITE intervention to support behavior change. [file 1471-2296-14-78-S1.docx]

| **PHASE** | **STRATEGY** |
| --- | --- |
| **Motivational phase**  *(Precontemplation)* | ***Provide knowledge*** about type 2 diabetes and its causes and effects  Make clear ***seriousness of***  type 2 diabetes  Explain ***extent of risk*** of participant for developing type 2 diabetes  Explain ***role of*** ***lifestyle*** (weight, diet, exercise) in developing diabetes  Help participant ***recognize*** role of ***own lifestyle*** in developing diabetes |
| **Motivational phase**  *(Contemplation)* | Stimulate ***action self-efficacy*** to overcome ***motivation action gap:***  Let participant think about ***pros and cons*** of changing behaviour  ***Discuss*** cons and ***emphasize*** pros  Emphasize that help is available, but ***participant decides*** |
| **Motivation-action gap**  *(preparation)* | ***Agenda setting***: let participant choose topic from project objectives  ***Ask questions***; what would participant regard as the problem?  Help participant to set ***SMART goals***  Help participant to think about possible solutions  Encourage participant to choose a ***concrete and realistic solution***  Help participant to develop a ***SMART action plan*** |
| **Action phase**  *(action)* | ***Evaluate*** lifestyle change with participant  If applicable: help the participant to ***define where’s the problem***;  topic, objectives, solution or action plan?  Help the participant to ***identify obstacles***  Enlarge ***coping self-efficacy*** to overcome obstacles  Give ***feedback***, provide ***trics and tools,***  discuss (immaterial) ***rewards*** and ***social support*** |
| **Action phase**  *(maintenance)* | Encourage participant to ***build habits*** of new behaviour  Help participant to identify future ***high-risk situations*** and to think ahead about possible solutions  Emphasize difference between ***lapse and relapse***  Help participant to make a ***plan*** for ***follow-up support*** |

Appendix 1: Overview of the theoretical framework of the APHRODITE intervention to support behavior change.
