# Supplementary material for: Behavior change in a lifestyle intervention for type 2 diabetes prevention in Dutch primary care: opportunities for intervention content
Source: BMC Fam Pract. 2013 Jun 7;14:78. doi: 10.1186/1471-2296-14-78 (PMC3706294; doi:10.1186/1471-2296-14-78)
Supplement: Additional file 2 — Planning of the APHRODITE intervention and content of the group consultations. [file 1471-2296-14-78-S2.docx]

| **Time** | **GP** | **NP *** | **Group-consultation** | **dietician** |
| --- | --- | --- | --- | --- |
| Baseline | Admission |  |  |  |
| Baseline |  | Admission |  |  |
| 1 month |  |  | *Topics:* nutrition components; calories and fat |  |
| 1 month |  |  | *Topics:* carbohydrates; sugar; sweeteners |  |
| 2 months |  |  |  | Consultation |
| 3 months |  | Follow-up |  |  |
| 6 months |  | Follow-up |  |  |
| 9 months | Follow-up |  |  |  |
| 9 months |  |  | *Topic:* exercise in relation to sugar metabolism |  |
| 12 months |  | Follow-up |  |  |
| 15 months | Follow-up |  |  |  |
| 15 months |  |  | *Topics:* food package labels; nutrition logo’s; fibres |  |
| 18 months |  | Follow-up |  |  |
| 21 months | Follow-up |  |  |  |
| 21 months |  |  | *Topics:* food packages; food game to recall information |  |
| 24 months |  | Follow-up |  |  |
| 27 months | Conclusion |  |  |  |
| 30 months |  | Conclusion |  |  |

Appendix 2: Planning of the APHRODITE intervention and content of the group consultations.

* NP= nurse practitioner
