# Supplementary material for: Behavior change in a lifestyle intervention for type 2 diabetes prevention in Dutch primary care: opportunities for intervention content
Source: BMC Fam Pract. 2013 Jun 7;14:78. doi: 10.1186/1471-2296-14-78 (PMC3706294; doi:10.1186/1471-2296-14-78)
Supplement: Additional file 3 — Mode of delivery of the GP and nurse practitioner training. [file 1471-2296-14-78-S3.docx]

Appendix 3: Mode of delivery of the GP and nurse practitioner training

Before the start of the study, all GPs and nurse practitioners received a two-evening directive instruction on the theoretical framework of the intervention and its translation into practice. The training was designed to learn health care providers how to support participants to progress through the stages of change [[7](#_ENREF_7)] .

First, more information was provided about the Transtheoretical Model. As depicted below, the concept of the stages of change was visualized as a mountain with the precontemplation and contemplation phases on the left side (unfreeze), the maintenance phase on the right side (freeze) and -as actual behavior change is the most difficult part- the preparation and action phases at the top. Furthermore, it was emphasized that relapse to earlier stages is common.

GPs and nurse practitioners were taught how to use a simple question to get an impression of the stage of change of a participant for a certain behavior (for example eat more dietary fibre):

***‘Are you currently trying to eat more dietary fibre?’***

□ No and I’m not planning to do so within the next half year (precontemplation) □ No, but I’m planning to start trying within the next half year (contemplation) □ No, but I’m planning to start trying within the next month (preparation) □ Yes, I’m trying to eat more dietary fibre, but for less than half a year (action) □ Yes, I’m trying to eat more dietary fibre for more than half a year (maintenance)

Second, GPs and nurse practitioners were taught how to use known behavior change techniques to guide participant progress through the phases (motivational interviewing, filling out decisional balance sheets, goal setting, developing action plans, barrier identification, relapse prevention) [[8](#_ENREF_8), [15](#_ENREF_15)]. An overview of the techniques used in each of the phases is provided in appendix 1.

In addition to this two evening directive instruction, all nurse practitioners received a five-evening course in Motivational Interviewing (MI)[[17](#_ENREF_17)]. They were taught how to guide participants through the process of behavior change by letting them investigate their ambivalence towards change. Central to the course were the following five MI-principles:

* Approach the participant with empathy * Turn participant resistance into behavior change strength * Support participant self-efficacy * Investigate and emphasize discrepancies between the behavior shown and the behavior desired * Accept participant choices: don’t argue or discuss decisions made

As part of the course, active roleplaying was performed to practice with the behavior change and MI-techniques. Furthermore, consultations with participants were audio-taped to get feedback and advice from other nurse practitioners and the MI course-leader.

GP’s and NP’s were invited once per year for meetings to discuss progress and receive additional guidance from the project management team.
