# Supplementary material for: Behavior change in a lifestyle intervention for type 2 diabetes prevention in Dutch primary care: opportunities for intervention content
Source: BMC Fam Pract. 2013 Jun 7;14:78. doi: 10.1186/1471-2296-14-78 (PMC3706294; doi:10.1186/1471-2296-14-78)
Supplement: Additional file 4 — Questionnaire behavioral change APHRODITE study. [file 1471-2296-14-78-S4.doc]

**ABOUT YOUR EXPERIENCES**

With this part of the questionnaire we would like to gain more insight into your experiences in the diabetes prevention project. Improving your diet, losing weight or increasing physical activity are not easy: we are curious which difficulties you are facing and how you are dealing with them. Your answers **do not affect your participation** in the diabetes prevention project. The answers you give in this part of the questionnaire ‘about your experiences’ will **not be sent to your GP or nurse practitioner.**

***The following questions are about losing weight.*** *A healthy weight descreases the risk for diseases. You feel more healthy, which makes you feel good. You can assess whether your weight is healthy by measuring your waist circumference or your body mass index (BMI; weight / length * length). (Source: The Netherlands Nutrition Centre Foundation)*

**Question 1a. Please indicate to what extent you agree with the following statements**

*‘For me the pros of losing weight do not outweigh the cons’*

□ □ □ □ □ □

I totally disagree I disagree Neutral I agree I totally agree I don’t know

‘*I don’t think I’ll be successful in losing weight’*

□ □ □ □ □ □

I totally disagree I disagree Neutral I agree I totally agree Not applicable

**Question 1b.** **If you are neither planning nor trying to lose weight, could you please**

**indicate the most important reason for not doing so?**

□ Not applicable, I am planning or trying to lose weight → **go to question 1C**

□ I am neither planning nor trying to lose weight, because …………….................................. ……………………………………………………………………………………………………………………………………………………………………………………………………………………………………………………………............................. → **go to question 2A**

**Question 1c. Have you started to change your behaviour in order to lose weight?** **Are you trying to lose weight?**

□ Yes

□ No...................................................................................................... → **go to question 2A**

**Question 1d. Do you manage to lose weight? Are your methods to lose weight successful?**

□ Yes

□ No

**Question 1e.** **Regardless of whether or not you have managed to lose weight, what has**

**been the main obstacle to losing weight?**

**The main obstacle to losing weight has been**..……………………………………………... ……………………………………………………………………………………………………………………………………………………………………………………………………………………………………………………………………………………………................

***The following questions are about eating more fibres.*** *Dietary fibres are important for proper bowel movement. It is thought that a diet rich in fibres increases insulin sensitivity, which in turn could decrease the risk of developing diabetes. Furthermore, a diet rich in fibres is important to prevent overweight. Vegetables, fruits, legumes and wholegrain cereals are especially rich in dietary fibres. (Source: The Netherlands Nutrition Centre Foundation)*

**Question 2a. Please indicate to what extent you agree with the following statements**

*‘For me the pros of eating more dietary fibres do not outweigh the cons,*

□ □ □ □ □ □

I totally disagree I disagree Neutral I agree I totally agree I don’t know

‘*I don’t think I’ll be successful in eating more dietary fibres’*

□ □ □ □ □ □

I totally disagree I disagree Neutral I agree I totally agree Not applicable

**Question 2b.** **If you are neither planning nor trying to eat more dietary fibres, could you please indicate the most important reason for not doing so?**

□ Not applicable, I am planning or trying to eat more dietary fibres → **go to question 2C**

□ I am neither planning or trying to eat more dietary fibres, because...................................... ……………………………………………………………………………………………………………………………………………………………………………………………………………………………………………………………............................. → **go to question 3A**

**Question 2c. Have you started to eat more dietary fibres? Are you trying to eat more dietary fibres?**

□ Yes

□ No...................................................................................................... → **go to question 3A**

**Question 2d. Do you manage to eat more dietary fibres? Are your methods to eat more dietary fibres successful?**

□ Yes

□ No

**Question 2e.** **Regardless of whether or not you have managed to eat more dietary fibres, what has been the main obstacle to eating more dietary fibres?**

**The main obstacle to eating more dietary fibres has been**..…..…………………………... ……………………………………………………………………………………………………………………………………………………………………………………………………………………………………………………………………………………………................

***The following questions are about eating less fat.*** *Dietary fats are important for your health. In a healthy diet the fat content is between twenty and forty percent of the total amount of calories. Diets rich in fat however easily lead to a disturbed energy balance, which in turn can increase the risk of becoming overweight. (Source: The Netherlands Nutrition Centre Foundation) .*

**Question 3a. Please indicate to what extent you agree with the following statements**

*‘For me the pros of eating less fat do not outweigh the cons’*

□ □ □ □ □ □

I totally disagree I disagree Neutral I agree I totally agree I don’t know

‘*I don’t think I’ll be successful in eating less fat’*

□ □ □ □ □ □

I totally disagree I disagree Neutral I agree I totally agree Not applicable

**Question 3b.** **If you are neither planning nor trying to eat less fat, could you please indicate the most important reason for not doing so?**

□ Not applicable, I am planning or trying to eat less fat → **go to question 3C**

□ I am neither planning or trying to eat less fat, because.......................................................... ……………………………………………………………………………………………………………………………………………………………………………………………………………………………………………………………............................. → **go to question 4A**

**Question 3c. Have you started to eat less fat? Are you trying to eat less fat?**

□ Yes

□ No...................................................................................................... → **go to question 4A**

**Question 3d Do you manage to eat less fat? Are your methods to eat less fat successful?**

□ Yes

□ No

**Question 3e.** **Regardless of whether or not you have managed to eat less fat, what has been the main obstacle to eating less fat?**

**The main obstacle to eating less fat has been**…………………..…………………………... ……………………………………………………………………………………………………………………………………………………………………………………………………………………………………………………………………………………………................

***The following questions are about eating less saturated fat****. The fat content of food products consists of a combination of both saturated and unsaturated fats; the proportion of both types of fat however differs between products. It is more healthy to choose food products low in saturated fats and trans fats as both have a negative effect on developing type 2 diabetes. (Source: The Netherlands Nutrition Centre Foundation) .*

**Question 4a. Please indicate to what extent you agree with the following statements**

*‘For me the pros of eating less saturated fat do not outweigh the cons’*

□ □ □ □ □ □

I totally disagree I disagree Neutral I agree I totally agree I don’t know

‘*I don’t think I’ll be successful in eating less saturated fat’*

□ □ □ □ □ □

I totally disagree I disagree Neutral I agree I totally agree Not applicable

**Question 4b.** **If you are neither planning nor trying to eat less saturated fat, could you please indicate the most important reason for not doing so?**

□ Not applicable, I am planning or trying to eat less saturated fat → **go to question 4C**

□ I am neither planning or trying to eat less saturated fat, because............................................ ……………………………………………………………………………………………………………………………………………………………………………………………………………………………………………………………............................. → **go to question 5A**

**Question 4c. Have you started to eat less saturated fat? Are you trying to eat less saturated fat?**

□ Yes

□ No...................................................................................................... → **go to question 5A**

**Question 4d Do you manage to eat less saturated fat? Are your methods to eat less saturated fat successful?**

□ Yes

□ No

**Question 4e.** **Regardless of whether or not you have managed to eat less saturated fat, what has been the main obstacle to eating less saturated fat?**

**The main obstacle to eating less saturated fat has been**……….…………………………... ……………………………………………………………………………………………………………………………………………………………………………………………………………………………………………………………………………………………................

***The following questions are about increasing physical activity.*** *In order to keep a healthy weight or to lose weight it is important to exercise regularly. Furthermore, regular physical activity improves your health and physical appearance and reduces stress. (Source: www.30minutenbewegen.nl)*

**Question 5a. Please indicate to what extent you agree with the following statements**

*‘For me the pros of increasing physical activity do not outweigh the cons’*

□ □ □ □ □ □

I totally disagree I disagree Neutral I agree I totally agree I don’t know

‘*I don’t think I’ll be successful in increasing physical activity’*

□ □ □ □ □ □

I totally disagree I disagree Neutral I agree I totally agree Not applicable

**Question 5b.** **If you are neither planning nor trying to increase physical activity, could you please indicate the most important reason for not doing so?**

□ Not applicable, I am planning or trying to increase physical activity → **go to question 5C**

□ I am neither planning or trying to increase physical activity, because.................................... ……………………………………………………………………………………………………………………………………………………………………………………………………………………………………………………………............................. → **go to question 6**

**Question 5c. Have you started to increase physical activity? Are you trying to increase physical activity?**

□ Yes

□ No...................................................................................................... → **go to question 6**

**Question 5d Do you manage to increase physical activity? Are your methods to increase physical activity successful?**

□ Yes

□ No

**Question 5e.** **Regardless of whether or not you have managed to increase physical activity, what has been the main obstacle to increasing physical activity?**

**The main obstacle to increasing physical activity has been**……….……………………... ……………………………………………………………………………………………………………………………………………………………………………………………………………………………………………………………………………………………................
